# Dietary Sodium Restriction Reduces Arterial Stiffness, Vascular TGF-β-Dependent Fibrosis and Marinobufagenin in Young Normotensive Rats

**DOI:** 10.3390/ijms19103168

**Published:** 2018-10-15

**Authors:** Yulia N. Grigorova, Wen Wei, Natalia Petrashevskaya, Valentina Zernetkina, Ondrej Juhasz, Rachel Fenner, Christian Gilbert, Edward G. Lakatta, Joseph I. Shapiro, Alexei Y. Bagrov, Olga V. Fedorova

**Affiliations:** 1 Laboratory of Cardiovascular Science, National Institute on Aging, NIH, 251 Bayview Blvd, Baltimore, MD 21224, USA; grigorovaju@gmail.com (Y.N.G.); wen.wei@nih.gov (W.W.); petrashevskayn@mail.nih.gov (N.P.); valentina.zernetkina@nih.gov (V.Z.); JuhaszO@grc.nia.nih.gov (O.J.); rachel.fenner@nih.gov (R.F.); cgilbert5@elon.edu (C.G.); lakattae@grc.nia.nih.gov (E.G.L.); aybagrov@gmail.com (A.Y.B.); 2 Joan C. Edwards School of Medicine, Marshall University, 1600 Medical Center Drive, Huntington, WV 25701, USA; ShapiroJ@marshall.edu

**Keywords:** high sodium chloride diet, tissue fibrosis, collagen, transforming growth factor beta, TGFβ1, vascular smooth muscle cells, cardiotonic steroids, marinobufagenin, Na/K-ATPase inhibitors, pulse wave velocity, aortic stiffness

## Abstract

High salt (HS) intake stimulates the production of marinobufagenin (MBG), an endogenous steroidal Na/K-ATPase ligand, which activates profibrotic signaling. HS is accompanied by a blood pressure (BP) increase in salt-sensitive hypertension, but not in normotensive animals. Here, we investigated whether HS stimulates MBG production and activates transforming growth factor-beta (TGF-β) profibrotic signaling in young normotensive rats, and whether these changes can be reversed by reducing salt to a normal salt (NS) level. Three-month old male Sprague–Dawley rats received NS for 4 and 8 weeks (0.5% NaCl; NS4 and NS8), or HS for 4 and 8 weeks (4% NaCl; HS4 and HS8), or HS for 4 weeks followed by NS for 4 weeks (HS4/NS4), *n* = 8/group. Systolic BP (SBP), pulse wave velocity (PWV), MBG excretion, aortic collagen 1α2, collagen 4α1 and TGF-β, Smad2, Smad3, Fli-1 mRNA, and total collagen abundance were measured at baseline (BL), and on weeks 4 and 8. Statistical analysis was performed using one-way ANOVA. SBP was not affected by HS (125 ± 5 and 126 ± 6 vs. 128 ± 7 mmHg, HS4 and HS8 vs. BL, *p* > 0.05). HS increased MBG (164 ± 19 vs. 103 ± 19 pmol/24 h/kg, HS4 vs. BL, *p* < 0.05) and PWV (3.7 ± 0.2 vs. 2.7 ± 0.2 m/s, HS4 vs. NS4, *p* < 0.05). HS8 was associated with a further increase in MBG and PWV, with an increase in aortic *Col1a2* 80%), *Col4a1* (50%), *Tgfb1* (30%), *Smad2* (30%) and *Smad3* (45%) mRNAs, and aortic wall collagen (180%) vs. NS8 (all *p* < 0.05). NS following HS downregulated HS-induced factors: in HS4/NS4, the MBG level was 91 ± 12 pmol/24 h/kg (twofold lower than HS8, *p* < 0.01), PWV was 3.7 ± 0.3 vs. 4.7 ± 0.2 m/s (HS4/NS4 vs. HS8, *p* < 0.05), aortic wall *Tgfb1*, *Col1a2*, *Col4a1*, *Smad2*, *Smad3* mRNAs, and collagen abundance were reversed by salt reduction to the BL levels (*p* < 0.05). HS was associated with an activation of TGF-β signaling, aortic fibrosis and aortic stiffness accompanied by an MBG increase in the absence of SBP changes in young normotensive rats. The reduction of dietary salt following HS decreased MBG, PWV, aortic wall collagen and TGF-β. Thus, HS-induced aortic stiffness in normotensive animals occurred in the context of elevated MBG, which may activate SMAD-dependent TGF-β pro-fibrotic signaling. This data suggests that a decrease in salt consumption could help to restore aortic elasticity and diminish the risk of cardiovascular disease by reducing the production of the pro-fibrotic factor MBG.

## 1. Introduction

High salt (HS) consumption is associated with increases in blood pressure (BP) and vascular stiffening by altering vascular smooth muscle cell (VSMC) function and producing arterial wall fibrosis in salt-sensitive hypertension and with advancing age [1,2,3,4,5,6]. Notably, dietary HS intake is positively correlated with a faster pulse wave, indicative of stiffer arteries, and arterial stiffening precedes the development of hypertension with aging [7,8,9]. In contrast, dietary salt restriction was accompanied by a reduction in pulse wave velocity (PWV), a measurement of arterial stiffness in elderly adult subjects with elevated blood pressure, indicating an association between salt intake and arterial stiffening [10]. The main finding of this study is that aortic fibrosis is highly correlated with the renal excretion of marinobufagenin (MBG). MBG, an endogenous steroidal Na/K-ATPase ligand and a representative of the class of the cardiotonic steroids (CTS), inhibits renal and vascular Na/K-ATPase, leading to natriuresis and vasoconstriction in salt-sensitivity [11,12,13,14,15]. The production of the natriuretic compound MBG by the adrenocortical cells [16] is stimulated by HS intake [17,18,19] and observed in volume-expanded states, such as preeclampsia, chronic kidney diseases, and resistant hypertension [20,21,22]. The administration of MBG to the rats at the doses mimicking physiological plasma MBG levels stimulated collagen production in myocardial tissue [21]. Ex vivo stimulation of aortic vascular smooth muscle cells with MBG increases the dose-dependent production of collagen-1 [22]. The signaling function of Na/K-ATPase, which involves inhibition by CTS, was discovered almost two decades ago by Xie and co-authors [23,24,25,26] The mechanism underlying the profibrotic effect of MBG may involve an activation of two major pro-fibrotic pathways, Fli1-dependent [21,22,27] and TGFβ-dependent signaling [Zhang Y et al., unpublished observation]. Notably, an HS diet is associated with a stimulation of TGFβ in the cardiovascular tissue of young normotensive rats [28,29,30]. 

Both clinical and experimental evidences support the development of salt-induced hypertrophy of the arterial wall in the absence of arterial pressure changes [31,32]. Previously, we demonstrated that HS intake induced vascular fibrosis via an MBG-dependent mechanism in the absence of a BP increase in normotensive young rats, and this remodeling was reversed by the immunoneutralization of MBG [32]. In this study, the administration of highly specific monoclonal anti-MBG antibody in vivo reduced aortic fibrosis and restored aortic relaxation in animals after prolonged HS intake [32]. The observed changes in vascular wall morphology in the absence of hemodynamic changes indicate that possible arterial stiffening is independent of BP, and that the pro-fibrotic factor MBG is responsible for the development of tissue fibrosis [32]. 

The aims of the present study are (i) to investigate whether an HS diet stimulates MBG production and aortic fibrosis in parallel with an activation of aortic TGFβ pro-fibrotic signaling; and (ii) whether these changes can be reversed by reducing HS intake to a normal salt diet in young normotensive Sprague–Dawley rats. We hypothesize that a decrease of dietary salt intake after prolonged HS consumption can reduce MBG excretion and reverse aortic stiffness in the central arterial system in normotensive rats.

## 2. Results

**Figure 1:**
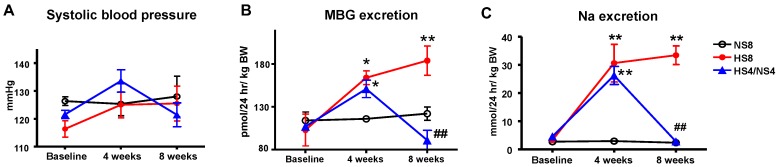
Effect of different salt regimens on systolic blood pressure (**A**); urine marinobufagenin (MBG) (**B**); and sodium excretion (**C**). Each point represents mean ± SEM, *n* = 8 rats per group. By one-way ANOVA: * *p* < 0.05, ** *p* < 0.01 vs. baseline; ^##^ *p* < 0.01 vs. HS 4 weeks. Experimental groups: NS8, normal salt diet for 8 weeks; HS8, high salt diet for 8 weeks; HS4/NS4, high salt diet for 4 weeks followed by 4 weeks of normal salt diet.

**Figure 2:**
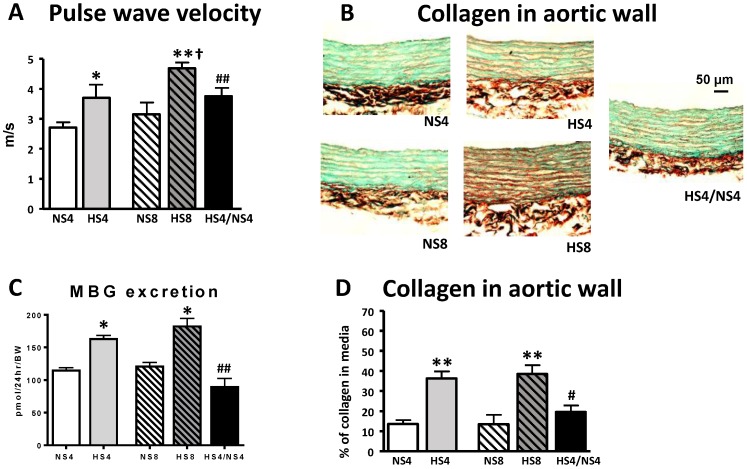
Effect of different salt regimens on aortic pulse wave velocity (**A**); aortic collagen content (**B**,**D**); and MBG (**C**) excretion in the different experimental groups. Total collagen was measured by Picro–Sirius Red/Fast Green stain; representative photomicrographs (**B**), and quantitative collagen content data (**D**) obtained from aortae derived from the different experimental groups. Bars presented as mean ± SEM; (**D**) data for collagen for the whole aorta were averages for each group, *n* = 8 rats per group. By one-way ANOVA followed by the Newman–Keuls test: * *p* < 0.05, ** *p* < 0.01 vs. normal salt diet at 8 weeks (NS8); ^#^ *p* < 0.05, ^##^ *p* < 0.01 vs. high salt diet at 8 weeks (HS8); ^†^ *p* < 0.05 vs. 4 weeks of normal salt diet (NS4). By two-way ANOVA: (**A**) effect of HS < 0.01, effect of age < 0.01, age-diet interaction is non-significant; (**C**,**D**) effect of HS < 0.01, effect of age and age-diet interaction are non-significant.

SBP did not change after 4 or 8 weeks of an HS diet (Figure 1A, Table 1). Urinary MBG excretion gradually increased during 8 weeks on an HS intake in parallel with the sodium excretion (Table 1, Figure 1B,C), and aortic PWV gradually increased on an HS diet (Figure 2A). PWV was significantly lower in the rats after dietary salt reduction (HS4/NS4 group) compared to HS8 (Figure 2A).

In the HS4/NS4 group, four weeks of HS intake was accompanied by an increase in PWV, MBG and sodium excretion, followed by a reduction of MBG and sodium excretion after four weeks of an NS diet (Table 1, Figure 1C and Figure 2A). FeNa was significantly higher after eight weeks of an HS diet in the HS8 group than in NS8 (Table 2). In the HS4/LS4 group, FeNa returned to the control level. Creatinine clearance did not change significantly in all groups (Table 2).

**Figure 3:**
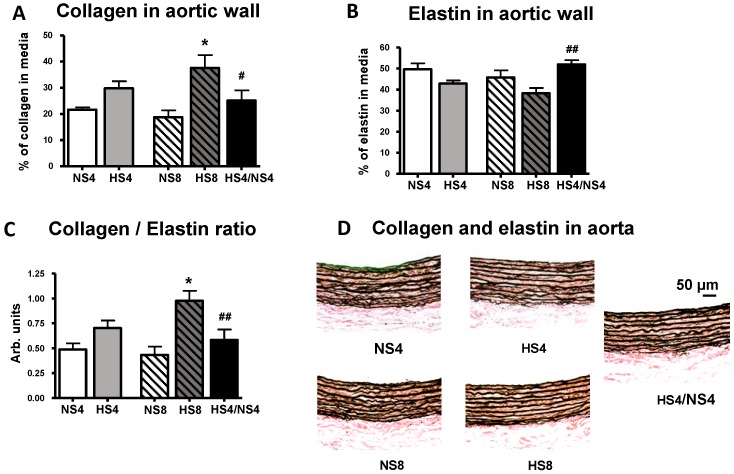
Images of elastin and collagen in the aortic wall by Verhoeff’s stain (collagen stained red, elastin stained black). Collagen (**A**), elastin (**B**), calculated collagen/elastin ratio (**C**) in aortic media and (**D**) representative photomicrographs of aortae from each experimental group are presented. Results are presented as mean ± SEM; data for collagen and elastin for the whole aorta were averages for each group, *n* = 8 rats per group. By one-way ANOVA followed by the Newman–Keuls test: * *p* < 0.01 vs. NS8; ^#^ *p* < 0.05, ^##^ *p* < 0.01 vs. HS8. By two-way ANOVA: (**A**,**C**) effect of HS < 0.01; effect of age and age-diet interaction are non-significant; (**B**) effect of HS < 0.05, effect of age < 0.05, effect of age and age-diet interaction are non-significant.

Figure 2 and Figure 3 show representative images and a comparison of collagen content and collagen/elastin ratio in the aortic wall between all groups. Both HS groups exhibited significantly higher collagen abundance compared to that in NS groups, in parallel with MBG and PWV changes (Figure 2).

Although elastin content was not significantly changed after HS intake, the collagen/elastin ratio was increased in the HS8 group, indicating an increased rigidity of the aortic wall. The dietary intervention in the HS4/NS4 group—i.e., HS followed by NS in 4-week increments—reduced the collagen abundance in the aortic wall and restored the collagen/elastin ratio, in parallel with MBG and PWV changes (Figure 2 and Figure 3).

**Figure 4:**
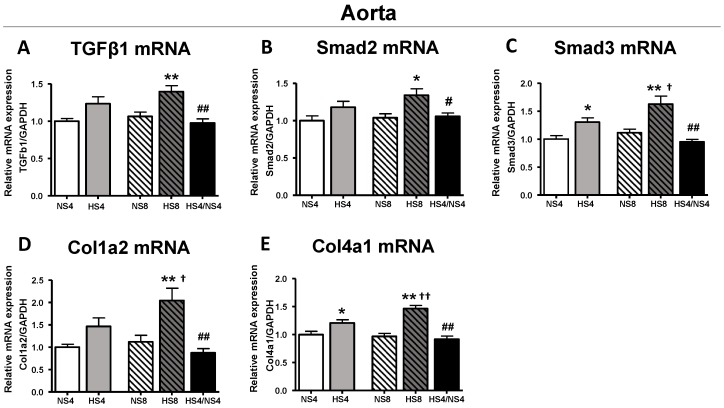
Effect of different salt dietary regimens on expression of transforming growth factor-beta (TGFβ1) (**A**); Smad2 (**B**); Smad3 (**C**); Col1a2 (**D**); and Col4a1 (**E**) mRNAs in aorta. Results are presented as mean ± SEM; *n* = 8 rats per group; for each sample, 4–5 measurements were performed in triplicates for each primer. By one-way ANOVA, followed by the Newman–Keuls test: * *p* < 0.05, ** *p* < 0.01 vs. NS4 or NS8; ^#^ *p* < 0.05, ^##^ *p* < 0.01 vs. HS8; ^†^ *p* < 0.05, ^††^ *p* < 0.01 vs. HS4. By two-way ANOVA: (**A**,**B**,**D**) effect of HS < 0.01; effect and age and age-diet interaction are non-significant; (**C**) effect of HS < 0.01, effect of age < 0.05, age-diet interaction is non-significant; (**E**) effect of HS < 0.01, effect of age is non-significant; age-diet interaction < 0.05.

Increased MBG levels, PWV and aortic wall collagen in the HS8 group were accompanied by a greater expression of aortic TGFβ1 (30%), Smad2 (30%), Smad3 (45%), collagen 1α2 (80%), and collagen 4α1 (50%) mRNAs (Figure 4). All these parameters were reversed after the restoration of the normal salt diet (Figure 2 and Figure 4).

**Figure 5:**
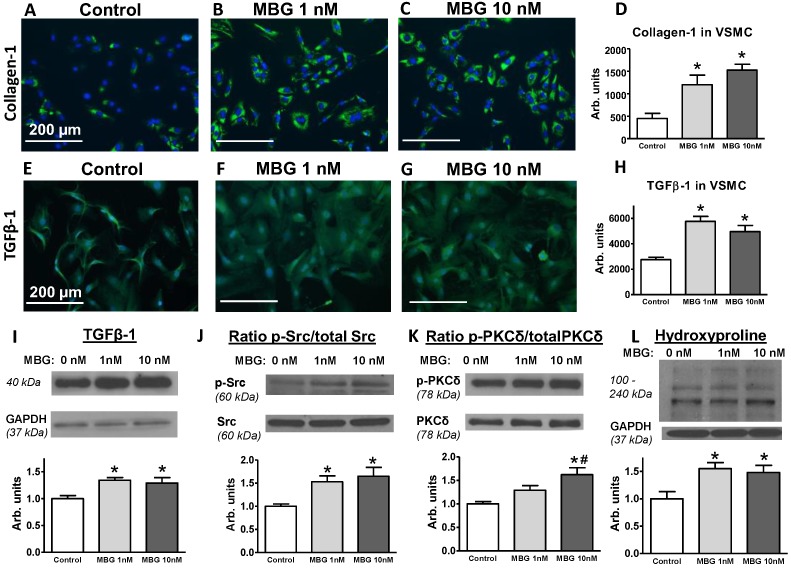
Collagen-1 and TGFβ-1 abundance by immunohistochemistry in the primary vascular smooth muscle cell (VSMC) culture, prepared from rat aortae (**A**–**H**). TGFβ-1 (**I**), ratio of phosphorylated Src to the total Src (**J**), ratio of phosphorylated PKCδ to the total PKCδ (**K**) and hydroxyproline (**L**) in the cultured VSMC (by Western blotting analysis). Cells were pretreated with a vehicle (control; no MBG), 1 nM and 10 nM of MBG for 24 h. Collagen-1 or TGFβ-1 was visualized with anti-collagen-1 antibody or with anti-TGFβ-1 antibody (green fluorescent) and nuclei counterstained with DAPI (blue) on the representative histochemistry images (**A**–**C**,**E**–**G**); Collagen-1 (**D**) and TGFβ-1 (**H**) in VSMC calculated as an area of green fluorescent stain per cell from 8–9 immunofluorescent images for each condition. (**I**–**K**), top panels: representative Western blotting images; bottom panels: statistical analysis of the band density for each protein standardized for GAPDH or the ratio of phosphorylated protein to the total protein abundance; each protein was tested 6–8 times for each condition. Results are presented as mean ± SEM. By one-way ANOVA, followed by the Newman–Keuls test: * *p* < 0.05, ^#^ *p* < 0.05 vs. control.

Stimulation of the cultured VSMC by 1 nM and 10 nM MBG was accompanied by increased collagen-1 (Figure 5A–D) and TGFβ-1 (Figure 5E–H) production by these cells compared to the control cells treated with the vehicle, i.e., no MBG. Western blotting analysis of the VSMC lysates revealed increased amount of matured form of TGFβ-1 (Figure 5I) and increased abundance of phosphorylated Src and PKCδ calculated to the total amount of the corresponding protein (Figure 5J,K). A lower dose of MBG (1 nM) upregulated TGFβ-1 and activated, i.e., phosphorylated Src. Pretreatment of the cells with 10 nM of MBG upregulated TGFβ-1 amount and increased the ratio of phosphorylated Src and PKCδ (Figure 5I–K). These findings indicated the activation of both TGFβ-1 and Src/PKCδ pathways by MBG in the rat VSMC. The hydroxyproline abundance was higher after the administration of both concentrations of MBG (Figure 5L). 

**Figure 6:**
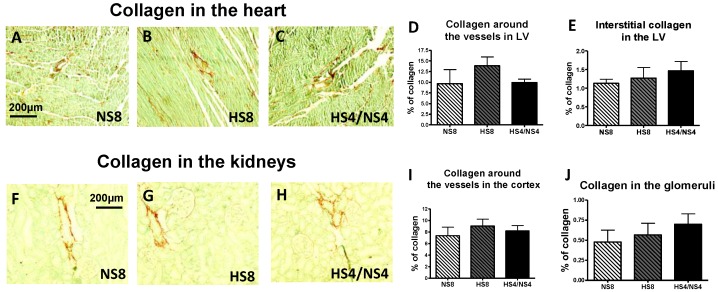
Collagen abundance in the heart (left ventricle; LV) and in the kidneys in normotensive rats of different salt regimens. Collagen content was measured around the small myocardial vessels (**D**) and in the interstitium (**E**) in LV and around small blood vessels in the cortex (**I**) and in the glomeruli (**J**) in the kidneys. Representative images of the LV from each group (**A**–**C**) and of the kidneys (**F**–**H**). Data presented as mean ± SEM and analyzed using one-way ANOVA; *n* = 8 rats per group, data from 3–4 separate fields were averages for each sample.

Collagen abundance in the left ventricle (LV) myocardium did not differ between all experimental groups, whereas there was a tendency of numerically higher collagen around the vessels of the LV from the rats on an HS intake. A reduction of the salt content in the diet showed a tendency towards a decrease in collagen level around the small myocardial arteries (Figure 6A–E). A similar observation was made for the kidneys: no statistical difference in the collagen levels was found in the interstitial medulla or cortex (data are not presented), and around the small vessels in the cortex or in the glomeruli (Figure 6F–J) between experimental groups.

## 3. Discussion

The present study demonstrates for the first time that dietary HS in normotensive rats is associated with unchanged SBP, the activation of arterial wall TGFβ signaling and an increased aortic stiffness in the presence of elevated level of Na/K-ATPase ligand MBG. Moreover, the rats exposed to a reduced salt diet (NS) after HS intake exhibited a decrease in MBG level, the inactivation of the pro-fibrotic TGFβ pathway, a decrease of aortic wall collagen abundance and the normalization of pulse wave velocity to control levels. Thus, MBG is a factor that links HS intake and pro-fibrotic signaling. This statement is also supported by our observation that MBG stimulates collagen production in parallel with activation of TGFβ-1 in cultured VSMC in vitro, i.e., in the absence of hemodynamic effects. Our present findings are in agreement with the findings of previous studies that HS stimulates MBG production [17,18,19,33,34], activates TGFβ signaling in vasculature [28,29], and that HS intake can be a pressure-independent event in normotensive young subjects [32,33,35,36,37].

MBG, a circulating endogenous Na/K-ATPase inhibitor, meets the requirements for a natriuretic hormone [4,11,38]. MBG is involved in the regulation of renal sodium transport, water–salt homeostasis and BP control. Inhibition of Na/K-ATPase in renal tubules promotes natriuresis, whereas at the scenario of renal insufficiency excessive MBG production would cause an inhibition of NKA in arterial vascular smooth muscle cells, which leads to an elevation of cytosolic Ca^2+^ and vasoconstriction via activation of an ionic pathway [39,40]. The enhanced production of MBG on an HS intake was described in the previous publications [19,33,34].

The present study involved young normotensive rats, which exhibited sufficient renal function able to sustain prolonged HS intake without BP increase. The exposure of the animals to an HS diet resulted in enhanced diuresis and natriuresis accompanied by a heightened MBG level (Figure 1, Table 1). A strong association between MBG excretion and urinary sodium in the rats on the different salt regimens indicates the utilization of the natriuretic function of MBG (Figure 1). HS intake was not associated with an impaired renal function in young animals, and salt withdrawal showed a decrease in MBG production and a return of the sodium excretion to the control level (Table 2), which is in agreement with our previous findings [10]. Notably, no significant changes in collagen deposition were observed in the renal cortex or medulla, and only a tendency for collagen increase around the small renal blood vessels was found in the cortex of the animals on an HS intake. Previous publications have documented an increase in the renal fibrosis in rats on an HS intake; the difference between our present study’s experimental setting and the settings in these publications are in the dietary salt amount (8% vs. 4% in our study) [30,36,41], or in the gender of the studied animals (females vs. males in the present study) [42].

In the present study, animals on an HS intake demonstrated an increased aortic PWV and collagen abundance in the aortic wall, an increased collagen/elastin ratio indicated on the augmented rigidity of aorta (Figure 2 and Figure 3), and an activation of the vascular pro-fibrotic TGF-β signaling (Figure 4). Previously, similar observations were made in normotensive rats on an HS diet [28,29]. The new finding in the present study is the association of the above changes with heightened MBG level on an HS diet, and that all these changes, including aortic fibrosis and PWV, were reversed by placing the animals on a NS diet after HS intake in four-week increments. Our previous clinical study demonstrated a similar positive correlation of MBG and PWV in the subjects with a sodium restriction intake after a habitual diet with a higher salt content [10]. Interestingly, our previous study, which was similar to the present experiment in the experimental setting, also revealed the connection of MBG and aortic fibrosis, because the aortic collagen abundance was reduced after the immunoneutralization of MBG with MBG-specific monoclonal antibody in vivo in the rats with an HS intake [32].

**Figure 7:**
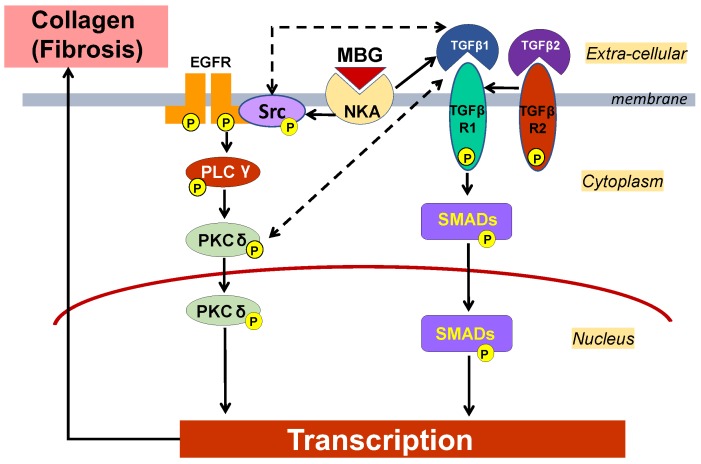
Scheme of the TGFβ SMAD-dependent pro-fibrotic pathway activated via binding of marinobufagenin (MBG) to Na/K-ATPase (NKA) (solid arrows). The interaction of MBG with NKA leads to a TGFβ-1 increase, which activates SMADs’ pro-fibrotic cascade. SMADs’ translocation to the nucleus results in an increase of transcription factors, which stimulates the production of pro-collagen and collagen. In addition, NKA inhibition by MBG initiates activation/phosphorylation of Src following by activation/phosphorylation PLCγ and PKCδ via scaffolding function of NKA [23,24,25,26,27,43]. Dotted arrows represent the complex relations between Src/PKCδ and TGFβ [44,45,46,47,48,49].

The mechanism of the pro-fibrotic pathway induced by MBG has been described in previous publications [21,22,27,43]. One of these pathways is Fli1-dependent signaling [21,22,27,43]. In these studies, the incubation of the vascular explants with MBG led to the degradation of Fli-1, a negative regulator of Col1 gene promoter, and resulted in increased collagen-1 synthesis [27,43]. In the present study, we observed the activation of TGFβ pro-fibrotic signaling in the presence of increased MBG in vivo in the animals on an HS intake, and in vitro in cultured VSMC stimulated with the physiological concentration of MBG (Figure 5). We also observed the activation/phosphorylation of Src and PKCδ proteins, which are related to the activation of several signaling pathways, including Fli1-dependent pro-fibrotic signaling. Previously, it was demonstrated that MBG and other CTS via binding to Na/K-ATPase phosphorylated, i.e., activated Src and PKCδ [27,43]. Numerous previous publications indicated the complex relations between Src/PKCδ and TGFβ [44,45,46,47,48,49] (Figure 7). HS intake initiates this signaling via the binding of MBG to Na/K-ATPase, and the activation of pro-fibrotic signaling may lead to reversible organ remodeling or initiate deep irreversible structural changes. Further studies of the MBG/Na/K-ATPase-initiated down-stream signaling (Figure 7) and the search for a pharmaceutical intervention to modulate these pathways merits future investigation.

In summary, this study demonstrated that HS intake was associated with an increase in Na/K-ATPase inhibitor MBG levels and an activation of the TGFβ1-mediated pro-fibrotic pathway in the vasculature, leading to an increase of aortic stiffness without elevation of BP. MBG activated TGFβ1 profibrotic pathway in cultured VSMC, suggesting a key role of MBG in the development of fibrosis via signaling function of Na/K-ATPase. The decrease in salt consumption restored the aortic elasticity via TGF-β pathway inactivation. Thus, lowering salt intake can improve vascular elasticity and decrease the risk of cardiovascular diseases by reducing the MBG level.

## 4. Methods

### 4.1. Animal Study

The experimental protocol was approved by the Animal Care and Use Committee of the National Institute on Aging, NIH (Animal Study Proposal 309, approved 11 January 2018). Male Sprague–Dawley rats, three months old, were maintained in a 26 °C environment with a 12:12-h light–dark cycle on an NS diet (0.5% NaCl; Harlan Teklad, Madison, WI, USA) and tap water ad libitum for adaptation for one week. The animals were divided into five groups: rats maintained on an NS diet for 4 weeks (NS4, *n* = 8) or for 8 weeks (NS8, *n* = 8); rats placed on an HS diet (4% NaCl, Harlan Teklad) for 4 weeks (HS4, *n* = 8) or for 8 weeks (HS8, *n* = 8); and the animals on an HS intake for 4 weeks followed by an NS diet for the next 4 weeks (HS4/NS4, *n* = 8). Body weights, systolic BP (SBP), heart rate and urine output were measured at baseline, at the end of weeks 4 and 8. SBP was recorded in the conscious rats by tail-cuff plethysmography (MRBP system, IITC Life Science, Woodland Hills, CA, USA). For urine collection, animals were placed in metabolic cages (Lab Products, Inc., Seaford, DE, USA) for 24 h, and urine aliquots were kept frozen for subsequent measurement of sodium, MBG, and creatinine (below). Aortic PWV was measured at weeks 4 and 8 of the experiment the next day following SBP measurement in the animals under anesthesia (see below). The rats were sacrificed by exsanguination from the abdominal aorta under deep anesthesia by Ketamine (100 mg/kg) and Xylazine (5 mg/kg). Blood, plasma, thoracic aortae, left ventricles (LV) and kidneys were collected. Aortic weight was expressed in milligrams per 100 g of body weight per mm of the total length. One half of the aorta, LV and kidney was fixed in 4% formalin buffer solution for the histochemical analysis.

### 4.2. Pulse Wave Velocity Measurement

The animals were placed under isoflurane (2.5% in oxygen) anesthesia to reduce stress and maintained on a heating pad in the supine position. The chest was shaved and electrocardiogram (ECG) leads were placed on two front legs and one rear leg, and ECG tracing was recorded. Aortic PWV was measured by the transit time method using a 12 MHz Doppler probe (Sonos 5500, Hewlett-Packard, Andover, MA, USA) at the transverse aortic arch and the abdominal aorta. The time at the transverse aortic arch (t_1_) and at the abdominal aorta (t_2_) was defined as the time from the peak of the ECG P wave to the foot of the velocity upstroke. The transit time (Δt) of the flow wave from the upper thoracic aorta to the lower abdominal aorta was determined as the time difference between two measurements (t_2_ − t_1_). The distance between two points of measurement was measured (d); the PWV was calculated as d/Δt. Each measurement of PWV and strain represents the average of five independent determinations per rat.

### 4.3. Blood Pressure Measurement 

SBP was recorded by tail-cuff plethysmography using MRBP system (IITC Life Science, Woodland Hills, CA, USA) in conscious animals at baseline, week 4 and week 8 of the experiment. The rats were habituated to the experimental environment by training to be restrained in a plastic tube with a tail cuff on for up to 30 min for two consecutive days. Every day the rats were restrained and placed in the chamber with 32 °C temperature maintained. After 5 min of the acclimation, five consecutive measurements were performed with 1-min interval. Then an animal was rested for 5 min, followed by five consecutive BP measurements. The measurement was repeated if necessary to obtain the stable readings. SBP and heart rate, performed on day 3, were used for the analysis. At each time point for each animal, 10–15 measurement of SBP were performed; the average of at least 5 stable readings was used.

### 4.4. Immunoassay

MBG concentration was measured using a DELFIA immunoassay kit based on a 4G4 monoclonal anti-MBG antibody [50]. The immunoassay is based on a competition between immobilized antigen (MBG-glycoside-Thyroglobulin) and MBG within the sample for a limited number of binding sites on 4G4 monoclonal anti-MBG (against MBG-glycoside-albumin) mouse antibody (1:500). Secondary anti-mouse antibody (1:4000, Perkin Elmer, Waltham, MA, USA) was labeled with non-radioactive europium. The cross-immunoreactivity of this antibody is (in %) MBG, 100; marinobufotoxin, 43; cinobufotalin, 40; telocinobufagin, 14; resibufagenin, 0.5; bufalin, 0.08; cinobufagin, 0.07; digoxin, 0.03; ouabain, 0.005; ouabagenin, 0.001; digoxigenin, 0.004; proscillaridin A, digitoxin, aldosterone, progesterone, prednisone, corticosterone, and thyroglobulin, <0.001. MBG (>98% HPLC-pure) was purified from secretions from parotid glands of Bufo marinus toads as reported previously [15]. Renal MBG excretion was expressed as pmol per 24 h per by body weight. 

### 4.5. Electrolytes in Plasma and Urine

Plasma levels of sodium and potassium were measured with i-STAT cartridges EC4+ (Abbott Point of Care, Princeton, NJ, USA). Urine sodium was measured using the Flame photometer (Bibby Scientific Limited, Staffordshire, UK), and the total excretion of sodium ions was expressed as mmol/24 h per kg of body weight.

### 4.6. Creatinine and FENa

Plasma creatinine level was measured using i-STAT cartridges Crea (Abbott Point of Care, Princeton, NJ, USA). Urine creatinine was measured by a colorimetric method using a commercial kit (Cayman Chemical, Ann Arbor, MI, USA). Urine and plasma creatinine and sodium were used for the calculations of creatinine clearance (CrCl) and fractional sodium excretion (FeNa):

CrCl = (urinary creatinine conc./plasma creatinine conc.) × 24 h urine volume/1440);

FeNa = urinary sodium conc. × plasma creatinine conc./(plasma sodium conc. × urinary creatinine conc.).

### 4.7. Histochemistry

The aortae, kidneys and LV were fixed in 4% buffered formalin and embedded in paraffin. After the removal of paraffin and rehydration, tissue sections (6 µm thick) were stained with Picro–Sirius Red/Fast Green (Chondrex, Inc., Redmond, WA, USA), and in the separate experiment aortae stained with Verhoeff Elastin Staining Kit (American Mastertech, Lodi, CA, USA). The slides were then dehydrated three times in 100% ethanol, cleared in xylene, and mounted with Permount (Fisher Scientific, Asheville, NC, USA). For quantitative image analysis, the MetaMorph 7.8.0.0 (Molecular Devices, Sunnyvale, CA, USA) was used. In aortic tissues, collagen and elastin was assessed in the media and expressed as percent of collagen in the media. In the LV, interstitial collagen was calculated as a percent of collagen to the selected area for each image, and the amount of collagen around the vessel was expressed as a percent to the total area of the vessel. Collagen in the kidneys was assessed separately in the cortex, glomeruli and around the small cortical vessels and expressed similarly to the LV.

### 4.8. Real-Time Quantitative PCR

Real-time quantitative analysis of TGFβ1, Smad2, Smad3, collagen 1α2, collagen 4α1 and Fli-1 mRNAs levels in aortic tissue was performed by PCR amplification of the resulting cDNAs and normalized to expression of housekeeping rat GAPDH genes as the internal standards. Total RNA was extracted from aortic tissues and then reverse transcribed to cDNA using the TaqMan Reverse transcription kit (Life Technologies/Applied Biosystems, Grand Island, NY, USA). Primers for real-time PCR for rat TGF-β1, SMAD 3 and collagen 1α2 genes, and housekeeping rat GAPDH genes were obtained from Qiagen (Qiagen Inc., Germantown, MD, USA). Quantitative real-time PCR was performed using QuantiFast SYBR Green PCR kit (Qiagen) according to the manufacturer’s protocol, and ABI 7300 Real-Time PCR System (Life Technologies/Applied Biosystems, Foster City, CA, USA). Gene expression of each sample was analyzed using the following protocol: activation at 95 °C (8 min) followed by 40 cycles, consisting of the first phase of denaturation at 95 °C (10 s), and the second phase of annealing/extending at 60 °C (30 s). Each reaction was performed in triplicate with the inclusion of no-template controls in each experiment. A dissociation curve analysis was performed in each experiment to eliminate non-specific amplification, including primer dimers. The GAPDH *C*_t_ values were subtracted from the raw sample *C*_t_ values to obtain the corrected *C*_t_. Power conversion (power (2^−(corrected *C*t^^)^)) was used to convert corrected *C*_t_ to relative RNA quantity.

### 4.9. Vascular Smooth Muscle Cell Culture

VSMC were prepared from the aortae obtained from six 4–6-month old male Sprague–Dawley rats according to previously published protocol [22] with minor modifications. Briefly, aortae were incubated with collagenase type I (Worthington Biochemical Corporation, Lakewood, NJ, USA) in Hank’s balanced salt solution (HBSS; Life Technologies, Grand Island, NY, USA) with antibiotic mixture (penicillin and streptomycin with an addition of antibiotic-antimycotic mixture; Life Technologies) at 37 °C for 30 min. Then adventitia and endothelial cells were removed, and the resulting tissues were incubated in DMEM (Life Technologies) with 10% FBS (fetal bovine serum; Life Technologies), and antibiotic mixture at 37 °C overnight. Next, the tissues were minced with scissors and incubated for 1.5 h with elastase (0.5 mg/mL; Sigma, St. Louis, MO, USA) and collagenase type II (Worthington Biochemical Corporation) in HBSS buffer with antibiotics. Digestion was stopped by adding DMEM with 10% FBS and antibiotics. The cells were filtered through a 70 μm cells strainer, collected by centrifugation (500× *g* for 5 min), and plated on collagen-covered tissue culture plate in DMEM with 10% FBS with antibiotics.

VSMCs prior to the 5th passage (~90% confluence) were depleted in FBS (1%) for 24 h followed by 24 h incubation with MBG (0, 1, or 10 nmol/L). After 24 h, the VSMCs were washed twice in PBS (phosphate buffered saline; Life Technologies), fixed with 4% paraformaldehyde (Sigma) in PBS for 20 min, permeabilized with 0.2% TritonX-100 (Sigma) for 15 min, were washed 3 times with PBS and blocked with 1% BSA (bovine serum albumin, Life Technologies) in PBS for 1 h. Some of the VSMC were used for Western blotting analysis (below). VSMCs were incubated with the primary rabbit polyclonal anti-collagen-1 antibody (1:250; Novus Biological, Littleton, CO, USA) or TGFβ-1 antibody (1:50, Santa Cruz Biotechnology, Inc., Santa Cruz, CA, USA) at 4 °C overnight. Next, VSMCs were incubated with the secondary fluorescent antibody (1:1000; Alexa Fluor 488 donkey anti-rabbit IgG (H+L); Life Technologies) for 1 h at room temperature, followed by 15 min incubation with 1µg/mL DAPI (4′,6-diamidino-2-phenylindole; Thermo Scientific, Waltham, MA, USA) for nucleus visualization. Immunofluorescent images were obtained by EVOS^®^ FL Cell Imaging System (Life Technologies). Collagen-1 and TGFβ-1 abundance as a green fluorescence intensity was measured by MetaMorph 7.8.0.0 (Molecular Devices) in each image (8–9 images for each condition) and presented as a fluorescence intensity per cell.

### 4.10. Western Blotting Analysis

The VSMC were homogenized in RIPA buffer (Santa Cruz Biotechnology, Inc.). Solubilized proteins were separated by 4–12% Tris-Glycine polyacrylamide gel electrophoresis and transferred to a nitrocellulose membrane (GE Health Care/Life Sciences, Pittsburgh, PA, USA). The proteins were visualized using rabbit polyclonal anti-TGFβ-1 (Cell Signaling Technology, Danvers, MA, USA; 1:500), anti-PKCδ and anti-phospho-PKCδ-Ser643/676 (Santa Cruz Biotechnology, Inc., 1:1000), anti-pan-Src and anti-phospho-Src (Tyr418) (Thermo Fisher Scientific Inc., Life Technologies Co., Carlsbad, CA, USA), and anti-hydroxyproline (Abcam, Cambridge, MA, USA; 1:50) antibodies, followed by incubation with peroxidase-conjugated anti-rabbit antiserum (GE Health Care/Life Sciences, 1:1000). Anti-hydroxyproline staining was assessed at the levels of the expected collagen-1, collagen-3 and collagen-4 localization (100 kDa–240 kDa). Bands were visualized by the Western blotting luminol reagent (Santa Cruz Biotechnology, Inc.). After 1–15 min exposure of nitrocellulose membrane on Premium Blue X-ray film (Phenix Research Products, Candler, NC, USA), the optical density was quantified by the laser densitometry (Kodak Molecular Imaging Software, version 5.0, Rochester, NY, USA). To normalize levels of proteins against levels of glyceraldehydes-3-phosphate dehydrogenase (GAPDH), membranes were stripped and re-probed with a rabbit monoclonal anti-GAPDH antibody (Cell Signaling Technology, 1:1000).

### 4.11. Statistical Analysis

Data were presented as mean ± standard error of the mean (SEM). For the statistical analysis, we used Graph Pad Prism 7 software (La Jolla, CA, USA). The data were analyzed using the *t*-test, one-way ANOVA, followed by a post-hoc test utilizing Newman–Keuls correction for multiple comparisons, and two-way ANOVA, followed by Bonferroni posttests. A two-sided *p* value of less than 0.05 was considered to be statistically significant.

## Figures and Tables

**Table 1:** Effect of different salt regimens on the physiological parameters in young male Sprague–Dawley rats.

	NS4 (*n* = 8)	NS8 (*n* = 8)	HS4 (*n* = 8)	HS8 (*n* = 8)	HS4/NS4 (*n* = 8)
Body weight (g)	472 ± 7	519 ± 13	514 ± 30	547 ± 17	553 ± 18
SBP (mmHg)	126 ± 4	128 ± 7	125 ± 5	126 ± 6	121 ± 4
Heart rate (beats/min)	336 ± 37	366 ± 8	342 ± 6	343 ± 9	345 ± 11
Urine volume (mL/kg BW)	23.8 ± 0.9	44.9 ± 5.1	62.8 ± 5.3 *	64.2 ± 11.6	27.6 ± 3.9 ^†^
Urinary Na (mmol/24 h/kg BW)	2.9 ± 0.4	2.3 ± 0.4	30.6 ± 6.6 *	33.4 ± 3.3 ^#^	2.8 ± 0.5 ^†^
Plasma Na (mmol/L)	141.0 ± 0.7	140.0 ± 0.2	141.0 ± 0.5	141.1 ± 0.5	140.2 ± 0.3
Plasma K (mmol/L)	4.5 ± 0.1	4.7 ± 0.1	4.4 ± 0.1	4.8 ± 0.2	4.5 ± 0.1
Aortic weights (g/mm/kg BW)	2.44 ± 0.08	2.58 ± 0.05	2.68 ± 0.12	2.86 ± 0.2	2.63 ± 0.1

Values are presented as mean ± SEM. The data were analyzed using one-way ANOVA, followed by the Newman–Keuls test to compare group pairs: * *p* < 0.01 vs. NS4; ^#^ *p* < 0.01 vs. NS8; ^†^ *p* < 0.05 vs. HS8. NS4, normal salt diet (0.5% NaCl) for 4 weeks; HS4, high salt diet (4% NaCl) for 4 weeks; NS8, NS diet for 8 weeks; HS8, HS diet for 8 weeks; HS4/NS4, HS diet for 4 weeks followed by 4 weeks of NS diet; SBP, systolic blood pressure; BW, body weight.

**Table 2:** Effect of different salt regimens on renal function in young male Sprague–Dawley rats.

	NS4 (*n* = 8)	NS8 (*n* = 8)	HS4 (*n* = 8)	HS8 (*n* = 8)	HS4/NS4 (*n* = 8)
Urinary Na, mmol/24 h	1.48 ± 0.21	1.29 ± 0.25	15.3 ± 3.2 **	18.4 ± 2.0 ^##^	1.57 ± 0.31 ^††^
Plasma Na, mmol/L	141.0 ± 0.7	140.0 ± 0.2	141.0 ± 0.5	141.1 ± 0.5	140.2 ± 0.3
Urine creatinine, mg/dL	196 ± 12	100 ± 17	76 ± 15 **	64 ± 11	217 ± 29 ^††^
Plasma Creatinine, mg/dL	0.33 ± 0.02	0.38 ± 0.03	0.35 ± 0.03	0.37 ± 0.02	0.37 ± 0.02
Urine volume, mL	11.3 ± 0.4	23.0 ± 2.6	31.8 ± 2.3 **	35.5 ± 6.9 ^##^	15 ± 1.9 ^††^
Creatinine clearance, mL/min	4.7 ± 0.4	4.1 ± 0.5	4.0 ± 0.9	4.22 ± 1.1	5.2 ± 0.4
FeNa, %	0.08 ± 0.03	0.15 ± 0.03	2.5 ± 0.51 *	3.16 ± 1.34 ^##^	0.15 ± 0.02 ^††^

Values are presented as mean ± SEM. The data were analyzed using one-way ANOVA, followed by the Newman–Keuls test to compare group pairs. * *p* < 0.05, ** *p* < 0.01 vs. NS4 weeks; ^##^ *p* < 0.01 vs. NS8; ^††^ *p* < 0.01 vs. HS8. Na, sodium; FeNa, fractional sodium excretion.
